# Antidiabetic Effect of Fermented *Mesembryanthemum crystallinum* L. in *db*/*db* Mice Involves Regulation of PI3K-Akt Pathway

**DOI:** 10.3390/cimb45080405

**Published:** 2023-08-03

**Authors:** Hye-Lin Kim, Yunu Jung, Hyo In Kim, Nak-Yun Sung, Min-Jee Kim, In-Jun Han, Geon Kim, Eun Yeong Nho, Sang-Yun Park, Yohan Han, Ji Hoon Jung, Dong-Sub Kim, Jinbong Park

**Affiliations:** 1 College of Korean Medicine, Kyung Hee University, Seoul 02453, Republic of Korea; 2 Division of Natural Product Research, Korea Prime Pharmacy Co., Ltd., Suwon 16229, Republic of Koreapobe1263@gmail.com (E.Y.N.); sy.park@koreaprime.co.kr (S.-Y.P.);; 3 Department of Surgery, Beth Israel Deaconess Medical Center, Harvard Medical School, Boston, MA 02115, USA

**Keywords:** type 2 diabetes, fermented ice plant, *Mesembryanthemum crystallinum* L., insulin receptor substrate 1, phosphoinositide 3-kinase, protein kinase B, AMP-activated protein kinase, nuclear factor erythroid 2–related factor 2

## Abstract

Type 2 diabetes (T2D) is a serious health issue with increasing incidences worldwide. However, current medications have limitations due to side effects such as decreased appetite, stomach pain, diarrhea, and extreme tiredness. Here, we report the effect of fermented ice plant (FMC) in the T2M mouse model of *db*/*db* mice. FMC showed a greater inhibition of lipid accumulation compared to unfermented ice plant extract. Two-week oral administration with FMC inhibited body weight gain, lowered fasting blood glucose, and improved glucose tolerance. Serum parameters related to T2D including insulin, glycosylated hemoglobin, adiponectin, and cholesterols were improved as well. Histological analysis confirmed the protective effect of FMC on pancreas and liver destruction. FMC treatment significantly increased the expression and phosphorylation of IRS-1, PI3K, and AKT. Additionally, AMP-activated protein kinase phosphorylation and nuclear factor erythroid 2–related factor 2 were also increased in the liver tissues of *db*/*db* mice treated with FMC. Overall, our results indicate the anti-diabetic effect of FMC; therefore, we suggest that FMC may be useful as a therapeutic agent for T2D.

## 1. Introduction

People in modern society consume excessively due to economic affluence and westernized eating habits. Combined with decreased physical activities, an increased incidence of chronic metabolic diseases such as obesity, hyperlipidemia, and diabetes are frequently seen [1,2]. Diabetes is classified into type 1 and type 2, the former being caused by loss of function of beta cells in the pancreas and the latter being mainly caused by insulin resistance and accompanying beta cell malfunction [3]. Type 2 diabetes (T2D) is more prevalent than type 1 diabetes is [4]. As described earlier, T2D leads to hyperglycemia due to various causes, and the main causes are insulin resistance, insulin secretion defects in beta cells, and excessive glucose production in the liver [5,6,7]. There is a report that T2D develops into vascular complications after 10 years if not actively managed from the onset [8]. According to a recent study, T2D causes oxidative stress due to damaged islet systems and excessive glycation [9,10,11].

Currently, T2D is mostly managed through diet, exercise, and weight control. Medications for T2D are classified into the following categories depending on the mechanism of action: insulin secretion stimulators (i.e., sulfonylurea), insulin sensitivity improvers, liver gluconeogenesis inhibitors (i.e., metformin), and glucose absorption inhibitors/glucosidase inhibitors (i.e., acarbose) with or without insulin injection [12,13,14]. However, the long-term use of these drugs causes side effects such as hypoglycemia, increased body fat, hepatotoxicity, and insulin resistance [15,16]. Accordingly, interest in natural products is increasing for the treatment and improvement of diabetes considering their safety. Recently, clinical studies suggest fenugreek seed extract [17], hookery extract, and rice bran oil [18] as natural products exerting anti-diabetic effects.

Ice plant (*Mesembryanthemum crystallinum* L.) is a succulent plant native to the Namib Desert in South Africa with a low germination rate and slow growth rate. It is mainly distributed in the dry regions of Africa, southwestern Australia, and western America. Hence, the name ‘ice plant’ is used because of the presence of ice crystal-like-shaped epidermal bladder cells on the stem and leaves [19]. Bladder cells contain pinitol, which lowers blood sugar levels, and myoinositol, which suppresses triglyceride levels, so it is known as a beneficial plant for diabetic patients [20,21,22]. In addition, its antibacterial [23], antioxidant [24], and memory improvement [25] effects have been reported. The ice plant has a great nutritional profile with a fresh salty, lemony flavor [26,27]. It has become popular in several countries including India, Australia, New Zealand, Germany and the United States as food in salad or as a beverage [20,28]. Current reports on ice plants mostly involve cultivation technology. Additionally, there is no available research regarding the effect of fermented ice plants. In this study, we use bioconversion technologies by applying the fermentation process to ice plants and seek to increase its effect on T2D.

## 2. Materials and Methods

### 2.1. Sample Purchase and Extract Preparation

**Figure 1:**
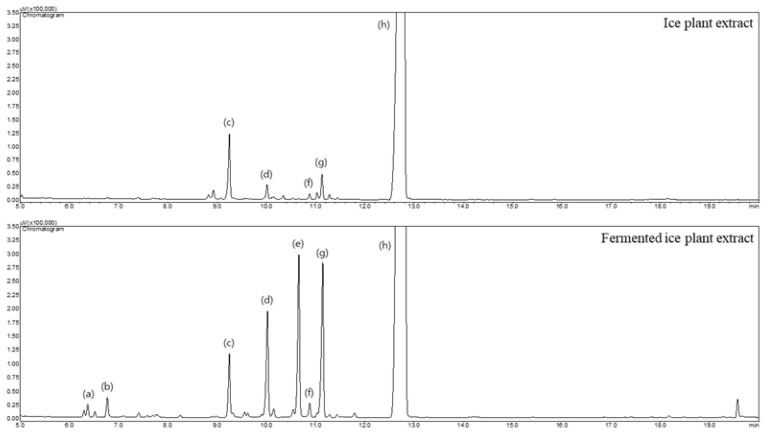
GC/FID chromatograms of ice plant medium and FMC. Peaks in (a) Arabinose, (b) Ribose, (c) D-Pinitol, (d) Glucose, (e) Mannitol, (f) D-chiro-inositol, (g) Galactose, and (h) myo-inositol.

Fermented ice plant extract (FMC) was provided from Korea Prime Pharmaceutical Co., Ltd. (Gwangju, Republic of Korea). The fermentation process produced various monosaccharides and inositols (Figure 1). Nutrient components of the FMC applied after the fermentation process were analyzed. Calories, total carbohydrate, total sugars, protein, total fat, saturated fat, trans fat, cholesterol, and sodium were confirmed (Supplemental Table S1).

### 2.2. Gas-Chromatographic Analysis

For monosaccharide and inositol analysis, 1 mL of fermented ice plant extract was taken in a 15 mL conical tube, and 9 mL of solution (H_2_O:C_2_H_5_OH = 1:1, *v*/*v*) was added and filtered with a 0.2 um syringe filter. Briefly, 200 uL of the filtrate was taken, and the extraction solution was evaporated until dry under a nitrogen gas flow. Then, 200 μL of trimethylsilylimidazole (TMSI) was added to the sample, shaken for 10 min and reacted for 30 min in a water bath at 80 °C for derivatization. At the end of the derivatization, the test solution was shaken again and centrifuged (10 °C, 8000 rpm, 5 min) to use as a test solution for GC/FID analysis. The GC/FID analysis was carried out using Shimadzu GC-2010AF plus (Shimadzu, Kyoto, Japan). He at 1 mL/min was used as a carrier gas. A HP-5 capillary column (0.25 mm i.d.× 30 m, 0.25 μm film thickness, J&W Scientific, Folsom, CA, USA) was used. The oven temperature was programmed as follows: the initial temperature of 150 °C was kept for 5 min, increased at a rate of 7.0 °C/min to 300 °C and maintained for 10 min. The injector and detector temperatures were set to 280 and 300 °C, respectively. Injections were made in the split mode, with a split flow of 1:10, and the injection amount of the sample was 1 μL.

### 2.3. Animal Experiments

All animal experiments were conducted in accordance with the Declaration of Helsinki, and approved by the ethical committee for the Animal Care of ChemOn Inc. (Suwon-si, Gyeonggi-do, Republic of Korea) protocol (approval number CHEM-2022-IA0237-00, date of approval 19 April 2022). Five-week-old C57BLKS/J-*db*/*db* mice (*db*/*db*; purchased from Saeron Bio Co., Ltd., Gyeonggi-do, Republic of Korea) were used as the T2D animal model. Mice were acclimatized for 1 week. A normal chow diet and water were fed ad libitum. Blood glucose was measured once a week for the following 3 weeks, and the mice were divided into 5 groups (*n* = 7 per group) based on blood sugar and body weight. For an additional 2 weeks, the control group (*db*/*db*) was treated with PBS, 3 groups were orally administrated with 100, 200, or 400 mg/kg/day of FMC, and the positive control group was treated with metformin (e.g., 250 mg/kg/day). Body weight and blood glucose were measured once a week. At the end of the experiment, mice were fasted overnight. The next day, body weight and fasting blood glucose were measured, and blood was collected from the heart after anesthesia with ethyl ether. Collected blood was placed in a 1.5 mL ep-tube and centrifuged at 3000 rpm at 4 °C for 30 min to obtain serum. After blood collection, liver, pancreas, and adipose tissue were separated and weighed. For the normal body weight mouse study, C57BL6 mice (Daehan Biolink, Chungbuk, Republic of Korea) were provided with a normal chow diet and water ad libitum. After acclimation, FMC at 100, 200 and 400 mg/kg of body weight was orally administered every day for 2 weeks. After overnight fasting, glucose and insulin were measured, and blood was collected for further serum analysis.

### 2.4. Oral Glucose Tolerance Test (OGTT)

To measure fasting glucose levels, an OGTT was performed. Briefly, mice were fasted for 12 h overnight on day 10 and then 2 g/kg of glucose was orally administered. Blood glucose was measured using a glucometer (gDoctor, AGM400, Allmedicus, Anyang-si, Gyeonggi-do, Republic of Korea) in blood collected from the tail vein right before (0 min) and 30, 60, 90, 120 min after glucose administration.

### 2.5. Biochemical Analysis of Blood Serum

Insulin, adiponectin, glycated hemoglobin (HbA1c), alanine transaminase (ALT), aspartate transaminase (AST), Creatinine, total cholesterol, TG, HDL, and LDL in the blood serum were analyzed by KP&T Co., Ltd. (Cheongju, Chungcheongbuk-do, Republic of Korea). Creatinine (ab65340) and blood urea nitrogen (BUN) (ab83362) were measured using kits purchased from Abcam Inc. (Cambridge, MA, USA).

### 2.6. Histological Analysis by Hematoxylin & Eosin (H&E) Staining and Immunohistochemical (IHC) Staining

H&E and IHC staining were performed for histological analysis. In brief, pancreatic and liver tissues were paraffin-embedded through dehydration and clearing as previously reported [29]. Then, 4 μm thick paraffin tissue sections were prepared with a microtome (Finesse ME Microtome, Thermo Shandon).

H&E staining was performed after the deparaffinization, hydration and water washing of the tissue section slides. The slides were incubated in hematoxylin for 10 min, washed, and then stained with eosin for 2 min. IHC staining was performed via the incubation of the slides with the primary antibody (1:100) overnight at 4 °C, followed by 3,3′-diaminobenzidine (DAB) staining for 3 min; then, hematoxylin staining was performed. All images were obtained under a regular light microscope and analyzed with Motic DSAssistant Software (Motic VM V1 Viewer 2.0, Hong Kong).

### 2.7. RNA Isolation and Real-Time Reverse Transcription-Polymerase Chain Reaction (RT-PCR)

RNA isolation and real-time RT-PCR procedures were performed as previously described [30]. *β actin* was used as the endogenous control. The primers used in this study are as follows:

*Irs1* (5′-CAAGGAGGTCTGGCAGGTTA-3′,
    5′-GGCCACGCGTCTGATATTC-3′)
*Pi3k* (5′-ACTTAGCTTCCGACACCACA-3′,
    5′-CACAGGAATGGCAAGGTAGC-3′)
*Pdk1* (5′-CTACATTAAGGCTCTGTCG-3′,
    5′-CAAATTTAGCAGAAACCACA-3′)
*Akt1* (5′-GCCTCTGCTTTGTCATGGAG-3′,
    5′-AGCATGAGGTTCTCCAGCTT-3′)
*Srebp1* (5′-CAGCAGGTCCCAGTTGTACT-3′,
    5′-GGTGGATGGGCAGTTTGTCT-3′)
*Glut2* (5′-ACAGACACCCCACTTACA-3′,
    5′-TCCTGATACACTTCGTCC-3′)
*G6pase* (5′-AAAAAGCCAACGTATGGATTCCG-3′,
    5′-CAGCAAGGTAGATCCGGGA-3′)

### 2.8. Western Blot Analysis

Protein extracts were prepared via homogenization in radioimmunoprecipitation assay (RIPA) buffer (Cell Signaling Technology, Danvers, MA, USA). Lysates were resolved via sodium dodecyl sulfate (SDS)-polyacrylamide gel electrophoresis and transferred onto polyvinylidene difluoride (PVDF) membranes (Millipore, Darmstadt, Germany). Then, the PVDF membranes were blocked and incubated with the indicated primary antibodies (1:1000), followed by incubation with horseradish peroxidase (HRP)-conjugated secondary antibodies (1:10,000). Protein signals were detected using the ECL advance kit (GE Healthcare Life Sciences, Jung-gu, Seoul, Republic of Korea).

### 2.9. Cell Culture

Briefly, 3T3-L1 mouse embryo fibroblasts were obtained from the American Type Culture Collection (ATCC, Rockville, MD, USA). Cells were maintained in DMEM containing 10% fetal bovine serum (Hyclone, Logan, UT, USA) with 100 units/mL of penicillin–streptomycin solution at 37 °C in 5% CO_2_ at 95% humidity until confluence. Two days after confluence (day 0), the cells were stimulated to differentiate with differentiation inducers (1 μM dexamethasone, 500 μM 3-isobutyl- 1-methylxanthine, and 1 μg/mL insulin) that were added to DMEM containing 10% FBS for 48 h. On day 2, 3T3-L1 cells were then cultured in DMEM containing 10% FBS supplemented with 1 μg/mL insulin, for another two days. At that time, MC and FMC were prepared in the differentiation medium at concentrations of 250 μg/mL, 500 μg /mL, and 750 μg /mL. Additionally, then, the cells were cultured in the 10% FBS/DMEM medium for an additional two days, at which time more than 90% of cells were mature adipocytes with accumulated fat droplets. On day 6, the cells were harvested and prepared for further experiments.

Human HepG2 cells were purchased from American Type Culture Collection. Cells were grown in DMEM containing 10% heat-inactivated fetal bovine serum and 100 units/mL of penicillin–streptomycin solution at 37 °C in 5% CO_2_. The medium was replaced every 2–3 days for all cell culture assays. MC and FMC were treated to HepG2 cells for 24 h prior to FFA exposure, at concentrations ranging from 10 to 100 μg/mL. Cells were incubated with 0.5 mM FFA composed of oleic acid (#O7501, Sigma-Aldrich Inc., Louis, MO, USA) and palmitic acid (#P0500, Sigma-Aldrich Inc., Louis, MO, USA) with or without MC and FMC (1–100 μg/mL) co-treatment for 24 h, and samples were harvested for analysis.

### 2.10. Oil Red O Staining

Briefly, the 3T3-L1 cells were seeded on 6-well plates. After the treatment, the cells were fixed with 10% formaldehyde at room temperature for 30 min. Subsequently, the cells were immersed in 60% isopropyl alcohol for 3 min, and dyed with 2 mg/mL of the Oil Red O (Sigma-Aldrich Inc., Louis, MO, USA) staining reagent for 60 min. After coloring, the cells were washed with ddH_2_O three times to clear the free dye. After microscopic image formation, intracellular triglycerides were quantified by detecting the amount of Oil Red O by adding 200 μL of isopropanol into each well. The optical density of extracted Oil Red O dye was measured at 500 nm using a microplate spectrophotometer (Bio-Rad, Hercules, CA, USA).

### 2.11. Statistical Analysis

Data are expressed as mean ± SD of independent experiments. Significant differences between groups were determined using the Mann–Whitney U test. All statistical analyses were performed using SPSS 11.5 (IBM SPSS, Chicago, IL, USA).

## 3. Results

### 3.1. Qualitative Analysis of Monosaccharides and Inositols

Figure 1 is a chromatogram analyzed via GC/FID after the derivatization of the FMC product with TMSI. The ice plant produced monosaccharides such as arabinose, ribose, glucose, and galactose, and inositols such as D-pinitol, mannitol, and D-*chiro*-inositol through a fermentation process. Among them, D-*chiro*-inositol, whose content increased through fermentation, and its methylated form, D-pinitol (3-O-methylchiro-inositol), are known to improve the efficiency of insulin use in the body. In this study, the content of D-*chiro*-inositol was increased by about three times after the fermentation process (Supplemental Figure S1).

### 3.2. FMC Decreases Blood Glucose and Body Weight Gain in db/db Mice

**Figure 2:**
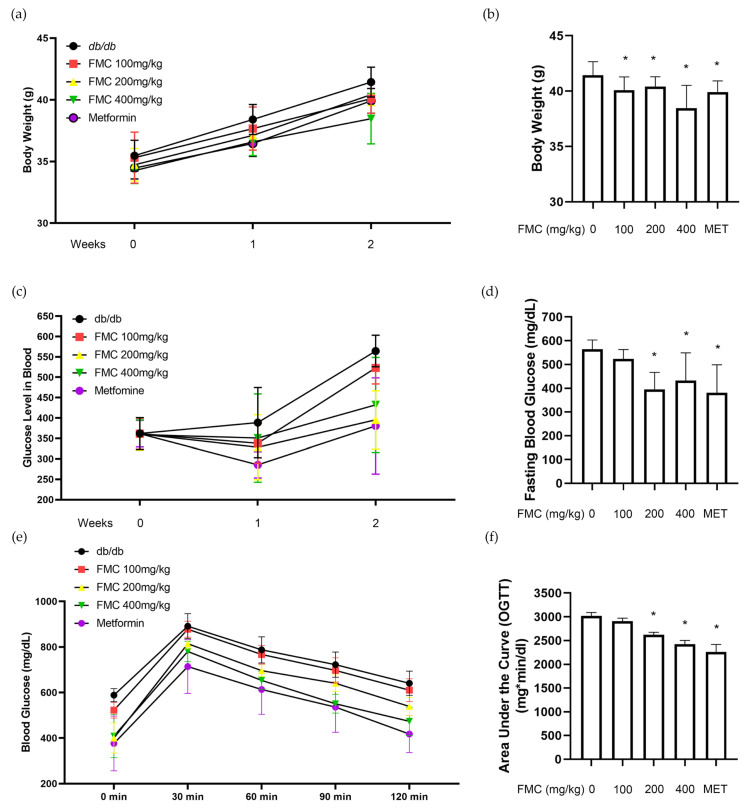
Effect of FMC on body weight, fasting blood glucose, and glucose tolerance in *db*/*db* mice. FMC was orally administrated for 2 weeks in *db*/*db* mice. After 2 weeks (**a**,**b**), the body weight of each group, (**c**,**d**) fasting blood glucose levels, and (**e**,**f**) glucose tolerance in *db*/*db* mice were measured with and without FMC administration. * *p* < 0.05 vs. *db*/*db* mice without FMC administration. Results are displayed as mean ± SD of n = 7 per group.

The use of the fermentation process was decided based on an Oil Red O staining assay of 3T3-L1 adipocytes, in which FMC showed a greater inhibition of lipid accumulation compared to the unprocessed extract (Supplemental Figure S2). To determine the administration dose and term for FMC, a pilot study in *db*/*db* mice was carried out. Mice were fed with 200, 400 and 600 mg/kg/day of FMC for 4 weeks. We could observe significant body weight loss and serum glucose decreases at week 2. There was no significant difference between 400 and 600 mg/kg/day (Supplemental Figure S3a,b). Additionally, FMC did not alter blood urea nitrogen or creatinine levels, indicating that no significant nephrotoxicity was induced by FMC at any dose (Supplemental Figure S3c). Zhang et al. reported that ice plant extract at a 400 mg/kg dose shows a positive effect on serum glucose levels [31]. Based on our pilot work and the previous literature, we selected 400 mg/kg as the highest dose, and set the administration term to 2 weeks to study the efficacy and mechanism of action of FMC. Two weeks following the administration of the test medication, the body weights of each experimental group of diabetic mice were as illustrated in Figure 2a,b. In the first week, week 0 of the experiment, there was no difference between all the experimental groups, but when FMC was taken orally for 2 weeks, substantial weight loss effects were confirmed at the dosages of 100, 200 and 400 mg/kg. In addition, it was confirmed that body weight was reduced in the positive control metformin group. The main characteristics of human T2D are not seen in C57BL/6J mice even though body weight is matched [32]. Hyperglycemia in type 2 diabetes is associated with the risk of macrovascular complications and has been reported as a cause of increased mortality [33,34]. Fasting blood glucose is shown in Figure 2c,d. When FMC was administered for 2 weeks, fasting blood glucose was lower than that of the control group by 29.9%, 23.4, and 23.6% in the 100, 200, and 400 mg/kg/day groups, respectively. Metformin group showed a 32.5% decrease. Results of the OGTT are shown in Figure 2e,f. Blood glucose at 120 min was 640.8 ± 23.88 mg/dL in the *db*/*db* mice, and in the FMC-administered group, blood glucose was significantly decreased in the 200 (539 ± 18.1 mg/dL) and 400 mg/kg groups (473.8 ± 27.65 mg/dL) (*p* < 0.05). In addition, the area under the curve (AUC), which indicates the value of the blood glucose elevation curve, confirmed that the FMC 200 and 400 groups showed improved blood glucose tolerance by 13.09% and 19.56% compared to that in the disease group. In normal mice treated with a normal chow diet, in other words in mice of normal body weight, FMC treatment did not induce any significant body weight change (Supplemental Figure S4a) or fasting glucose and insulin (Supplemental Figure S4b). Therefore, FMC significantly showed improved glucose tolerance in addition to suppressions of body weight gain.

### 3.3. FMC Improves T2D-Related Serum Parameters in db/db Mice

**Figure 3:**
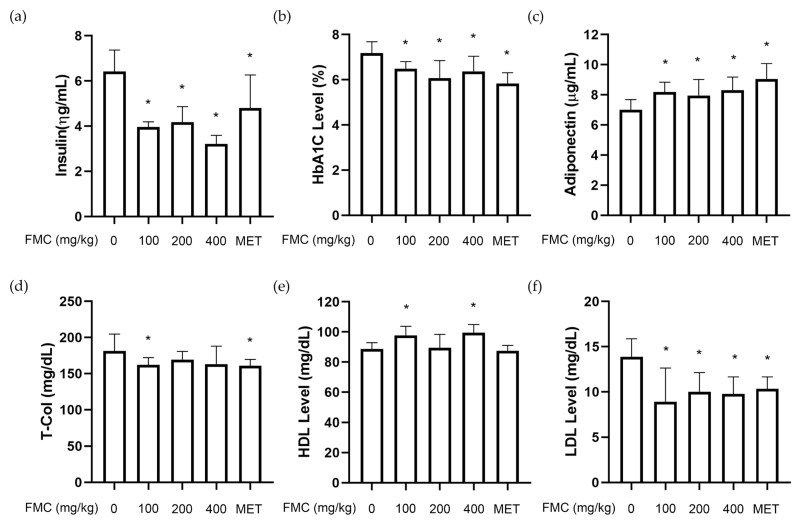
Effect of FMC on serum parameters in *db*/*db* mice. The (**a**) insulin, (**b**) HbA1c, (**c**) adiponectin, (**d**) T-Chol, (**e**) HDL, and (**f**) LDL levels in serum were measured. * *p* < 0.05 vs. *db*/*db* mice without FMC administration. Results are displayed as mean ± SD of n = 7 per group.

Hyperinsulinemia occurs when blood sugar levels rise, insulin action in the body is not carried out, and insulin resistance does not manifest as a result of impaired insulin function [35]. Glycated hemoglobin is one of the major signs when diagnosing diabetes, and its level is reported to increase by two to three times compared to that of the normal group [36]. It has been observed that a 1% drop in the level of glycated hemoglobin reduces the mortality rate caused by diabetes by 21% and the risk of micro-vascular complications by 37% [37]. Adiponectin is a hormone released by fat cells that increases insulin sensitivity in relation to blood sugar. It has been noted that the concentration of adiponectin falls when insulin resistance occurs [38]. As a result, it serves as an index to show how well blood sugar is controlled [39,40]. The findings of this study show the changes in blood insulin levels, glycated hemoglobin (HbA1c), and adiponectin levels induced by FMC treatment (Figure 3a–c). Briefly, the blood insulin concentration of the FMC 400 mg/kg/day-treated group was 4.15 ± 0.29 ηg/mL, while the *db*/*db* mice showed a significantly lower insulin level of 6.41 ± 0.42 ηg/mL. The HbA1c level was 6.36 ± 0.27% in the FMC 400 group, which is a significant decrease of 1.42% compared to that of the disease control group. The adiponectin level in the *db*/*db* group was 7.01 ± 0.29 μg/mL, but the FMC 100, 200, and 400 mg/kg/day groups showed a significant increase to 8.18, 7.95, and 8.43 μg/mL, respectively. T2D is closely related to obesity and can lead to dyslipidemia. Common symptoms of dyslipidemia include an increase in serum LDL and total cholesterol and a decrease in HDL levels. Plasma total cholesterol was 181.79 mg/dL in the *db*/*db* mice which significantly decreased to 162.33 mg/dL in the FMC 400 mg/kg group, demonstrating the inhibitory impact of FMC on hyperlipidemia (Figure 3d). The FMC 100 and 200 mg/kg treatment groups did show a tendency to decline, but there was no significant difference. However, there was a substantial decrease in LDL by 4.09 ± 0.14 mg/dL in the FMC 400 mg/kg group compared to that in the disease group, and a significant increase in HDL of 10.81 ± 0.58 mg/dL (Figure 3e,f). Therefore, we could confirm that FMC is efficient in reducing hyperlipidemia and blood sugar via biochemical analyses.

### 3.4. FMC Prevents Histopathological Changes and Increases Insulin Secretion in the Pancreas of db/db Mice

**Figure 4:**
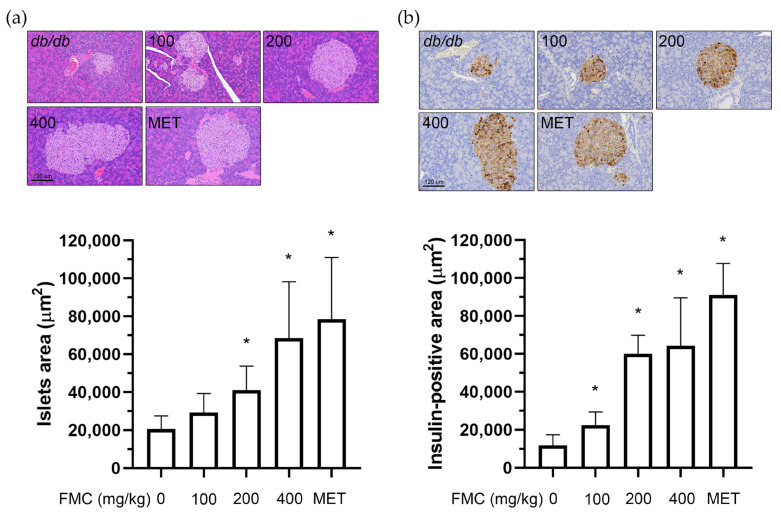
Effect of FMC on histopathologic analysis and insulin secretion in pancreas of *db*/*db* mice. (**a**) H&E-stained pancreas sections. A significantly smaller mean islet area was observed in *db*/*db* mice treated with FMC. (**b**) The distributions of insulin-positive cells in pancreas evaluated via IHC staining in *db*/*db* mice. * *p* < 0.05 vs. *db*/*db* mice without FMC administration. Results are displayed as mean ± SD of n = 7 per group.

Insulin regulates blood sugar and is distributed throughout the pancreatic Langerhans islet, while glucagon, which acts in an opposite manner to insulin, is distributed in the border of these islets [41]. When diabetes is induced, morphological abnormalities of islets within pancreatic tissue and a decrease in insulin secretion have been identified [42]. Therefore, the evaluation of the morphology, size, and insulin secretion activity of islets within pancreatic tissue is recognized as a crucial factor for the treatment of diabetes [43,44]. Langerhans islets in the normal group are distributed sparsely and are clearly distinguished from the exocrine part of the pancreas (Figure 4a). However, it is reported that the size decreases in *db*/*db* mice, and the shape of the ellipse appears irregularly [45]. A histological study was conducted to examine histological changes. H&E staining of the pancreas Langerhans islet and the islet area is shown in Figure 4a. In the FMC 200 and 400 mg/kg administration groups, an increase in islet sizes was observed. Further IHC staining on insulin immunoreactive cells showed that a greater number of stained cell areas were present in the FMC 200 and 400 mg/kg groups that in *db*/*db* mice (Figure 4b).

### 3.5. FMC Restores T2D-Induced Liver Injury in db/db Mice

**Figure 5:**
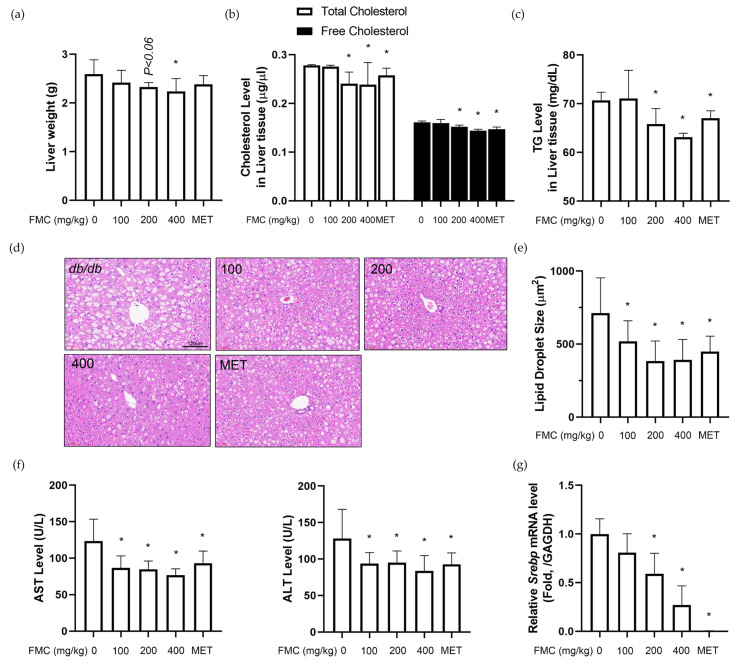
Effect of FMC on diabetic liver injury in *db*/*db* mice. (**a**) Significant reduction in weight due to FMC administration in *db*/*db* mice. (**b**,**c**) Markedly lower hepatic TG and TC levels in the livers of *db*/*db* mice treated with FMC. (**d**,**e**) Representative pictures of H&E-stained liver sections and measured lipid droplet size in liver tissue. (**f**) FMC administration in *db*/*db* mice showing less liver injury than vehicle-treated *db*/*db* mice based on serum ALT and AST levels. (**g**) Hepatic *Srebp* mRNA expression levels of genes associated with lipid metabolism measured via qRT-PCR. * *p* < 0.05 vs. *db*/*db* mice without FMC administration. Results are displayed as mean ± SD of n = 7 per group.

The liver was removed after autopsy and weighed to validate the effect of FMC on fat metabolism in the liver tissue. The weight of the liver tissue was significantly reduced by 13.68% in the FMC 400 mg/kg/day group (Figure 5a). Furthermore, the amount of fat in the liver tissue following FMC therapy in *db*/*db* mice was quantified (Figure 5d,e). Next, the levels of total cholesterol, free cholesterol, and TG were measured. FMC significantly decreased these blood parameters compared to those ien the *db*/*db* group. The positive control metformin also showed similar results (Figure 5b,c). We then determined the pathological changes in the liver tissue, in particular focusing on histology. In diabetic patients, lipid metabolism is impaired, and thus fat accumulation in the liver tissue is increased [46]. Our study showed similar results; the liver tissue of the *db*/*db* group had altered lipid accumulation. However, via FMC treatment, the lipid droplets were decreased (Figure 5d,e). The serum levels of AST and ALT were also determined to confirm the reduction in fatty liver (diabetic liver injury) due to FMC treatment (Figure 5f). No significant change in AST or ALT due to FMC administration was observed in normal-body-weight mice (Supplemental Figure S4c). *Srebp* is an important transcription factor of sterol regulatory element-binding protein (SREBP), which regulates lipid metabolism and the synthesis of cholesterol, FFA, and TG [47]. We measured the mRNA level of *Srebp1* in the liver tissue (Figure 5g). Results indicated a dose-dependent decrease in hepatic *Srebp1* due to FMC treatment.

### 3.6. FMC Increases Expression of IRS-PI3K-AKT Pathway in Liver Tissues of db/db Mice

**Figure 6:**
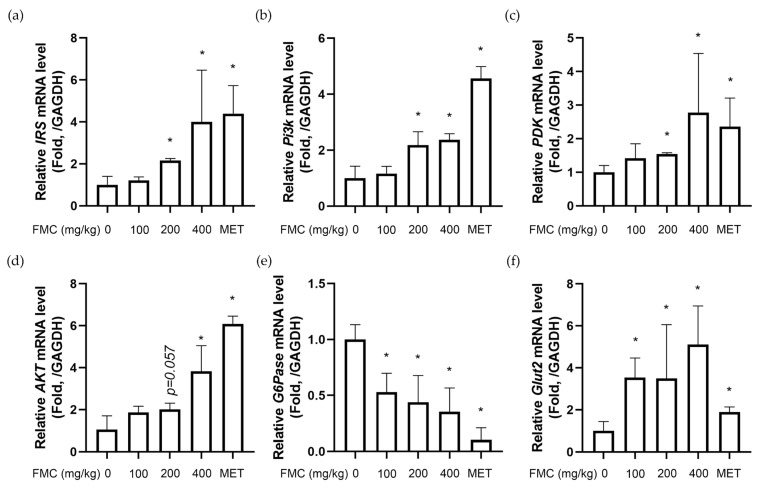

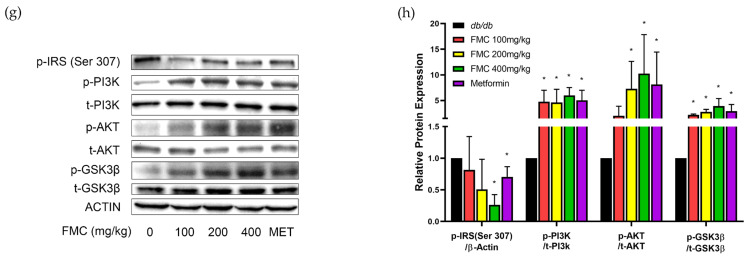
Effect of FMC on the IRS-PI3K-AKT pathway in liver tissues of *db*/*db* mice. (**a**–**f**) Hepatic *IRS*, *pi3k*, *PDK*, *AKT*, *G6Pase* and *Glut2* mRNA expression levels of genes associated with IRS signaling measured via qRT-PCR. (**g**,**h**) Representative pictures showing the protein levels of IRS, PI3K, AKT and GSK3β as confirmed via Western blot analysis. * *p* < 0.05 vs. *db*/*db* mice without FMC administration. Results are displayed as mean ± SD of n = 7 per group.

To better understand the mechanism of FMC, we evaluated the T2D-related mRNA expressions. In the FMC 400 mg/kg- and metformin-treated mice, the insulin receptor-related *Irs1*, *Pi3k*, *Pdk*, *AKT*, and *Glut2* levels were all significantly increased, and the mRNA level of *G6pase* was decreased (Figure 6a–f). IRS-1 has serine and tyrosine phosphorylation sites, in which the phosphorylation of Ser307 inhibits insulin signaling and lowers thyroxine phosphorylation levels. In general, IRS-1^Ser307^ phosphorylation is increased while the phosphorylation of PI3k, AKT, and glycogen synthase kinase-3 beta (GSK3β) is decreased in *db*/*db* mice [48]. Our results demonstrated that FMC treatment inhibited IRS-1^Ser307^ phosphorylation in a dose-dependent manner. On the contrary, the phosphorylation of PI3K, AKT, and GSK3β, as downstream factors of IRS-1, were increased by FMC (Figure 6g,h). Similarly, the IRS-PI3K-AKT pathway was altered by FMC treatment in a cell study using HepG2 cells (Supplemental Figure S5). Such results suggest that FMC can increase glucose uptake through the regulation of the IRS-PI3K-AKT pathway.

### 3.7. FMC Induces AMP-Activated Protein Kinase (AMPK) Phosphorylation and NRF2 Expression in Liver Tissues of db/db Mice

**Figure 7:**
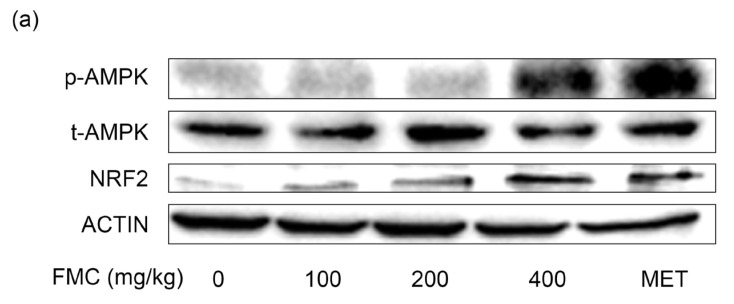

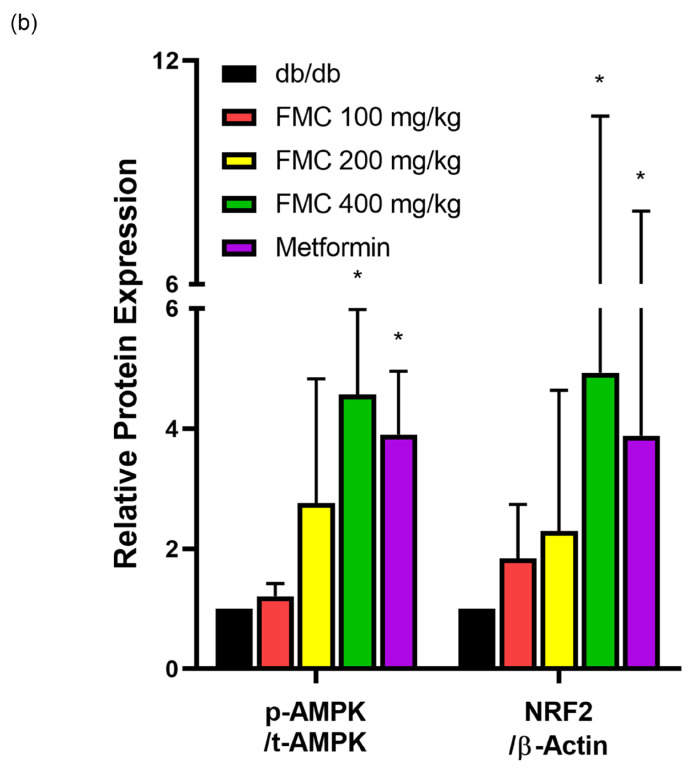
Effect of FMC on AMPK phosphorylation and NRF2 expression in liver tissues of *db*/*db* mice. (**a**,**b**) Representative pictures show the protein levels of AMPK and NRF2 as confirmed via Western blot analysis. * *p* < 0.05 vs. *db*/*db* mice without FMC administration. Results are displayed as mean ± SD of n = 7 per group.

Next, we investigated the expression of AMPK and NRF2. NRF2 works as a downstream marker of AMPK [49], and the phosphorylation of AMPK enhances Glut2 translocation [50] and lipid metabolism [51]. FMC administration significantly increased AMPK phosphorylation and NRF2 expression. AMPK and NRF2 were also significantly increased by metformin, which is well-known as an AMPK activator [52]. FMC 400 mg/kg and metformin administration increased AMPK phosphorylation by 4.5 and 3.9 times, respectively, and NRF2 expression increased it by 4.9 and 3.8 times compared to that in the *db*/*db* group (Figure 7a,b). These results suggest that FMC can increase glucose uptake through inducing AMPK phosphorylation and NRF2 expression.

## 4. Discussion

Ice plant is a natural source of phenolic compounds such as catechin, pyrogallol, vanillic acid, ellagic acid, and gallic acid, as well as D-pinitol, myo-inositol, and chiro-inositol. It is expected to be effective at treating T2D as it contains components with various functions [53]. In particular, the hypoglycemic control effect of D-chiro-inositol contained in the ice plant has been reported [54].

However, there is no study on the anti-diabetic effect of the ice plant yet. Even more, there is no corresponding study on FMC regarding its anti-diabetic features. Thus, we sought a bioconversion of this plant referring to Yoshida’s method [55] with an optimized enzyme treatment and fermentation process to maximize its effect. Through this fermentation process, the anti-adipogenic effect was enhanced; therefore, we decided to use FMC instead of the original plant extract.

*db*/*db* mice display T2D-like symptoms such as hyperglycemia, insulin resistance, and dyslipidemia. Therefore, this mouse strain lacking leptin receptors is widely used as an animal model for T2D research [56]. We measured blood glucose by orally administering FMC to confirm the hypoglycemic effect of FMC, and determined its effect on glucose metabolism through OGTT. Additionally, we observed that FMC administration significantly decreased glucose levels compared to the disease group and increased glucose metabolism. Through this, we could conclude that FMC improves glucose metabolism.

When blood glucose levels increase after eating, beta cells in the pancreas secrete insulin. Insulin enhances the uptake of glucose into cells and uses it as an energy source or converts it into glycogen and fatty acids [57]. However, in T2D patients, decreased insulin secretion from the pancreas, increased β-cell apoptosis, and decreased β-cell weight appear [58]. We confirmed the amount of insulin secretion in blood and pancreas upon FMC administration. FMC administration increased the insulin concentration in the blood, and we also showed that the reduced islet size and insulin secretion in the pancreas were reversed via FMC treatment.

Suppressed insulin secretion contributes to hyperglycemia by increasing the production of glucose in the liver. Insulin resistance is usually seen in the liver tissue of a T2D patient, and further increases glucose production even during fasting and postprandial periods. Such a process leads to liver hyperglycemia [59,60]. In T2D, liver fatty acid levels are elevated, which promotes fat storage in liver tissue. Therefore, 70% of T2D patients eventually develop non-alcoholic fatty liver diseases [61,62]. In addition, type 2 diabetes reduces insulin action and secretion, which results in the decreased inhibition of lipolysis in adipose tissue, raising blood fatty acid levels and increasing the delivery of fatty acids to the liver [61]. As a result, T2D individuals often have excessive liver fat accumulation and increased insulin resistance, which cause a malfunction of hepatic fatty acid metabolism, exacerbating hyperglycemia. Here, we report increased fat metabolism in the liver tissue via FMC administration. After administering FMC for 2 weeks, a significant decrease in fat accumulation in the liver tissue was observed. Additionally, cholesterol and triglyceride concentrations were decreased. Such results confirm that fat accumulation in the adipose tissue was reduced. SREBP-1C is a factor closely associated with fat synthesis in liver tissue. Reports indicate its high expression especially in the liver tissue of lipodystrophic mice [63]. A high expression of SREBP-1C results in increased fatty acids in the bloodstream and impaired insulin action in the liver [64]. We further confirmed that FMC administration decreased the expression of *Srebp1c*, suggesting that FMC can reduce fat accumulation by inhibiting fat synthesis in liver tissue.

We investigated the insulin signaling cascade to confirm the mechanism of FMC. The PI3k/AKT pathway is known to be an important regulator of insulin resistance [65]. Glucose transport into the liver is initiated by the phosphorylation of IRS-1 through an insulin-dependent mechanism. Subsequently, the phosphorylation of PI3k and phosphorylation of AKT takes place, and for the last step, GSK3β is phosphorylated. The activation of PI3K is known to inhibit gluconeogenesis [66]. The phosphorylation of IRS-1^Ser307^ is known to inhibit PI3k/AKT by reducing thyroxine phosphorylation and thus inhibiting insulin signaling [67]. Previous studies have shown that *db*/*db* mice have reduced IRS-1 protein expression in the liver, accompanied by reduced PI3k/AKT protein expression [68]. Here, we confirm that the phosphorylation of IRS-1^Ser307^ is reduced via FMC administration, and the phosphorylation of PI3K and AKT is increased in liver tissue. In addition, we observed that the phosphorylation of PI3K, AKT and GSK3β is increased in Hepg2 cell (Supplemental Figure S5). These results confirm that FMC has an anti-diabetic effect by restoring the function of the insulin signaling pathway.

AMPK plays an important role in glucose and fat metabolism in vivo, and abnormalities in AMPK are highly associated with metabolic diseases, cardiovascular disease, and cancer [69]. The activation of AMPK inhibits fatty acid synthesis and cholesterol synthesis. This promotes the process of producing ATP [70]. AMPK is closely related to the function and biology of the liver and pancreas. The activation of AMPK in the liver inhibits fatty acid and cholesterol synthesis and promotes fatty acid oxidation [71]. On the other hand, AMPK in pancreatic β cells is another regulator of insulin secretion [72]. Therefore, metformin or AICAR (both AMPK activators), are currently used for the treatment of T2D [73,74]. We confirmed that FMC administration increased AMPK phosphorylation and NRF2 expression (Figure 6a,b). Metformin, the currently available and a widely used drug for T2D, was used as a positive control. Our results demonstrate that FMC can play a role similar to that of metformin.

Importantly, side effects may occur as a result of pharmacological interventions. Thus, we conducted an in vivo study using normal mice with no pathological symptoms including diabetes to verify the rather unexpected effects of FMC with a healthy status. Two-week oral administration with FMC 100, 200 and 400 mg/kg did not induce any body weight change in mice fed with a normal chow diet (Supplemental Figure S4a). The fasting glucose and insulin levels were unaltered (Supplemental Figure S4b). Additionally, AST and ALT remained unchanged (Supplemental Figure S4c), as well as did the renal toxicity parameters creatinine and blood urea nitrogen levels (Supplemental Figure S4d). These results in normal-body-weight mice indicates that FMC does not show liver or kidney toxicity at the treated doses, and also does not induce unexpected metabolic changes in a healthy subject.

Our results demonstrated significant efficacy at doses of 200 mg/kg and 400 mg/kg of FMC, with a particularly improved effect observed at 400 mg/kg compared to 200 mg/kg. In our preliminary study (Supplemental Figure S3), we applied 200–600 mg/kg doses of FMC to mice. We could see a significant improvement in all dose groups from week 2, and the effect was dose-dependent. However, in week 3’s data, we found out that 400 and 600 mg/kg did not show statistical differences; thus, we concluded that the therapeutic ceiling lied in between the two doses. From the second in vivo experiment, from which came the main results of this study, we decided to use 100, 200, and 400 mg/kg of FMC. We selected three doses according to the FDA guidance of the therapeutic range. In our results, we can see that FMC exerts an anti-diabetic effect starting from a 100 mg/kg dose, but the maximal effect dose is 400 mg/kg. These findings serve as crucial evidence for establishing dosage settings in future clinical efficacy studies and emphasize the importance of high-dose administration. We expect the dose-dependent effect to come from the content of myo-insitol, D-pinitol, chiro-insitol and mannitol in FMC, and our further study should focus on which component shows the most effective anti-diabetic effect.

This study demonstrated the effect of FMC on T2D in vivo using *db*/*db* mice. FMC significantly inhibited weight gain, suppressed blood glucose, and improved OGTT results. Additionally, FMC decreased β cell damage and increased insulin sensitivity in the pancreas. Liver analysis showed that FMC treatment suppresses lipid accumulation while inhibiting cholesterol, TG, ALT, AST, *Srebp1* levels. Such an effect was seen through the alteration of the IRS/PI3K/AKT pathway and AMPK activation. Overall, we confirm that FMC can regulate glucose uptake through the activation of the IRS/PI3k/AKT and AMPK pathways in T2D mice.

## Data Availability

The data presented in this study are available on request from the corresponding author. The data are not publicly available due to an ongoing patent application.

## Supplementary Materials

The following supporting information can be downloaded at: https://www.mdpi.com/article/10.3390/cimb45080405/s1. Figure S1: Content of D-chiro-inositol after fermentation of iceplant extract. Figure S2: Effect of FMC and non-fermented ice plant extract (MC) on lipid accumulation in 3T3-L1 adipocytes. Figure S3: Effect of FMC administration on food intake in db/db mice. (a) Body weight change and (b) serum glucose levels were measured weekly. (c) Serum levels of blood urea nitrogen (BUN), creatinine, alanine aminotransferase (ALT), aspartate aminotransferase (AST), triglyceride (TG), total cholesterol, high-density lipoprotein (HDL) cholesterol and low-density lipoprotein (LDL) cholesterol were measured after 4 weeks of study. Figure S4: Effect of FMC on body weight, fasting blood glucose and insulin and serum parameters in normal mice. FMC was orally administrated for 2 weeks in normal mice. After 2 weeks (a) Body weight of each group, Fasting blood glucose levels and Fasting blood insulin levels (b) BUN, (c) Creatinine, (d) ALT and (e) AST levels in serum (d) in normal mice. were measured with and without FMC administration. * *p* < 0.05 vs. Normal mice without FMC administration. Results are displayed as mean ± SD of n = 5 per group. Figure S5: Effect of FMC treatment on the IRS-PI3K-AKT pathway in HepG2 cells. Representative pictures show the protein levels of IRS, PI3K, AKT and GSK3β as confirmed by western blot analysis. # *p* < 0.05 vs. vehicle-treated cells; * *p* < 0.05 vs. FFA-treated cells. Results are displayed as mean ± SD of n = 3 or more. Table S1: Nutritional contents of FMC.
