# Supplementary material for: Antidiabetic Effect of Fermented Mesembryanthemum crystallinum L. in db/db Mice Involves Regulation of PI3K-Akt Pathway
Source: Curr Issues Mol Biol. 2023 Aug 3;45(8):6415–31. doi: 10.3390/cimb45080405 (PMC10453056; doi:10.3390/cimb45080405)
Supplement: Supplementary file 1 [file cimb-45-00405-s001.zip › cimb-2451893-supplementary.pdf]

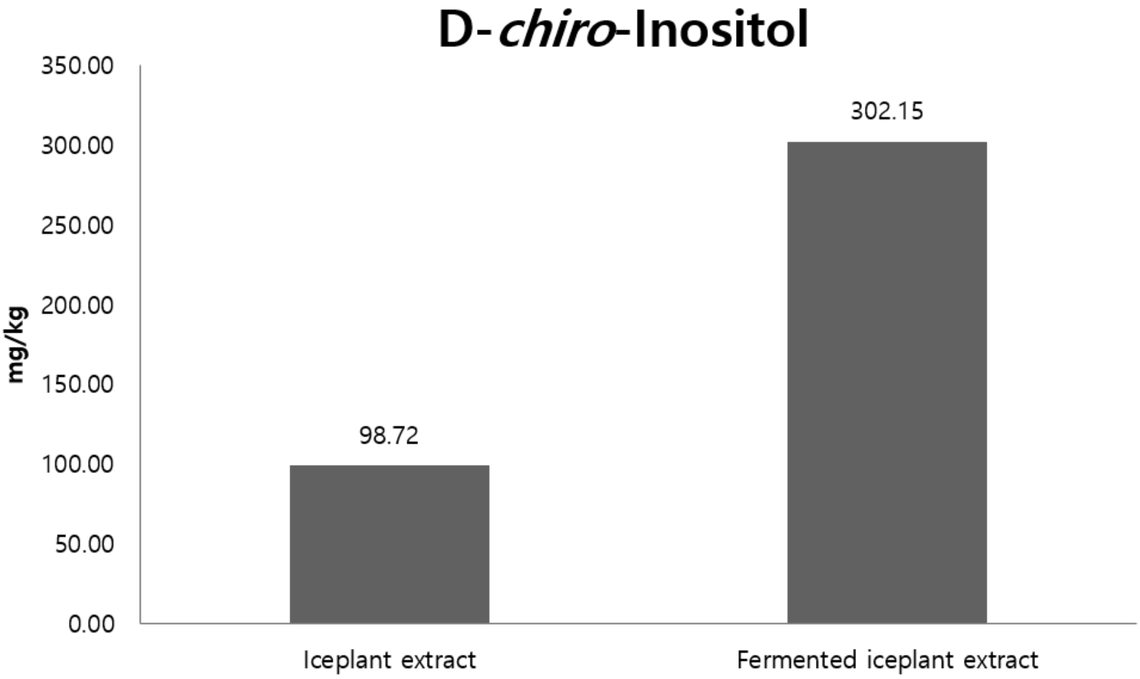

Supplemental Fig 1. Content of D-*chiro*-inositol after fermentation of iceplant extract.

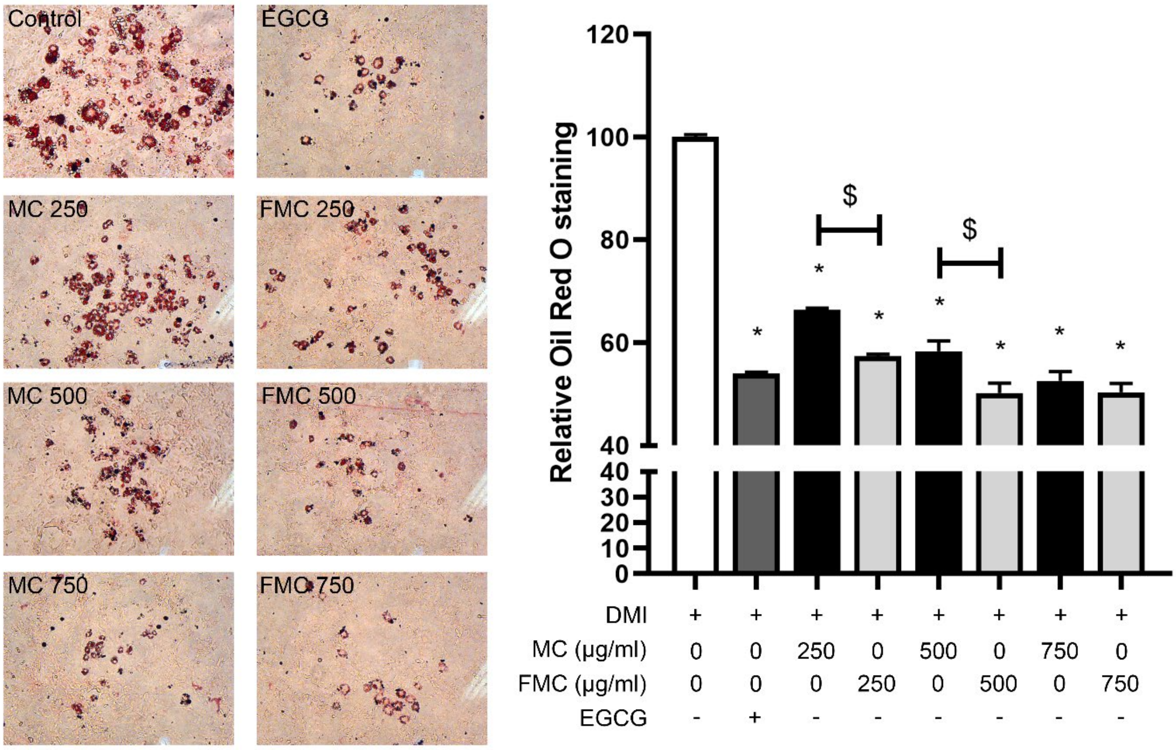

Supplemental Fig 2. Effect of FMC and non-fermented ice plant extract (MC) on lipid accumulation in 3T3-L1 adipocytes. *$p < 0.05$ vs. differentiated 3T3-L1 adipocytes; $p < 0.05 between indicated groups. Results are displayed as mean ± SD of n = 3 or more.

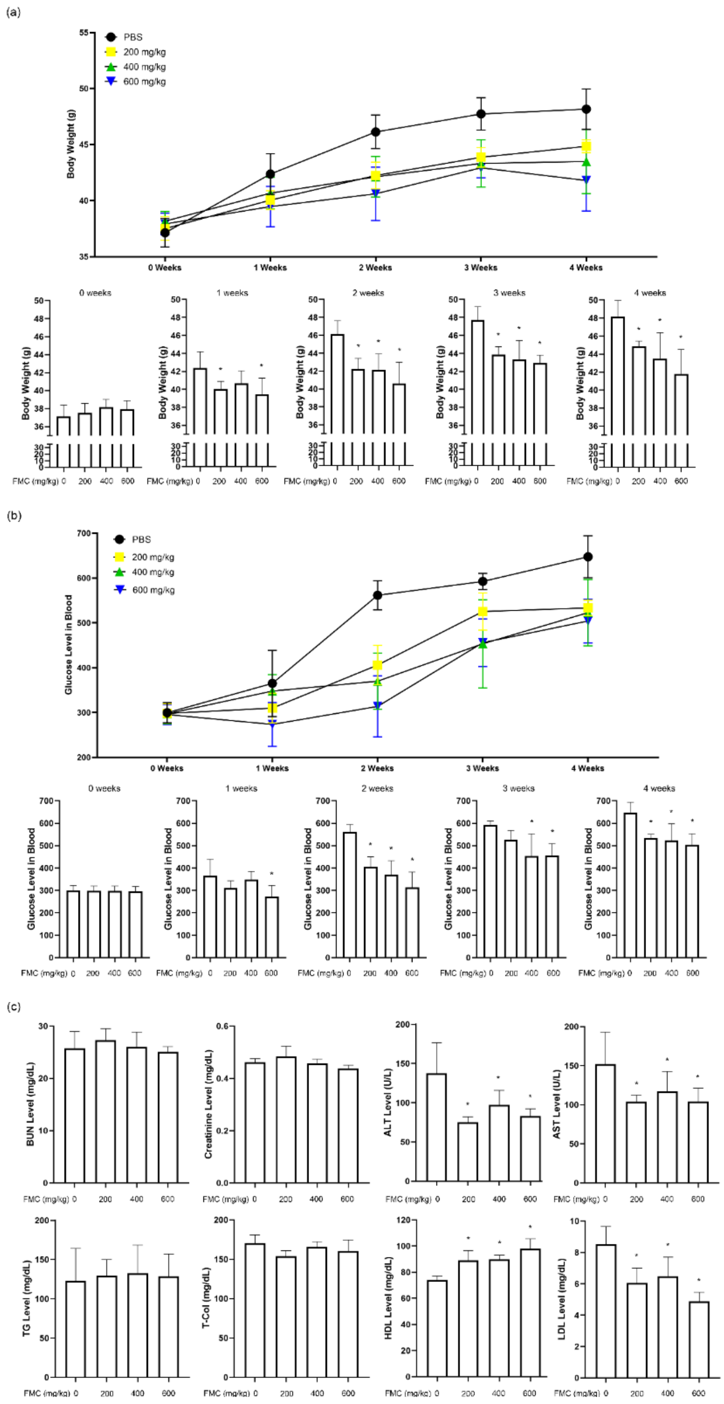

Supplemental Fig 3. Effect of FMC administration on food intake in *db/db* mice. (a) Body weight change and (b) serum glucose levels were measured weekly. (c) Serum levels of blood urea nitrogen (BUN), creatinine, alanine aminotransferase (ALT), aspartate aminotransferase (AST), triglyceride (TG), total cholesterol, high-density lipoprotein (HDL) cholesterol and low-density lipoprotein (LDL) cholesterol were measured after 4 weeks of study. *$p < 0.05$ vs. *db/db* mice without FMC administration. Results are displayed as mean ± SD of n = 7 per group.

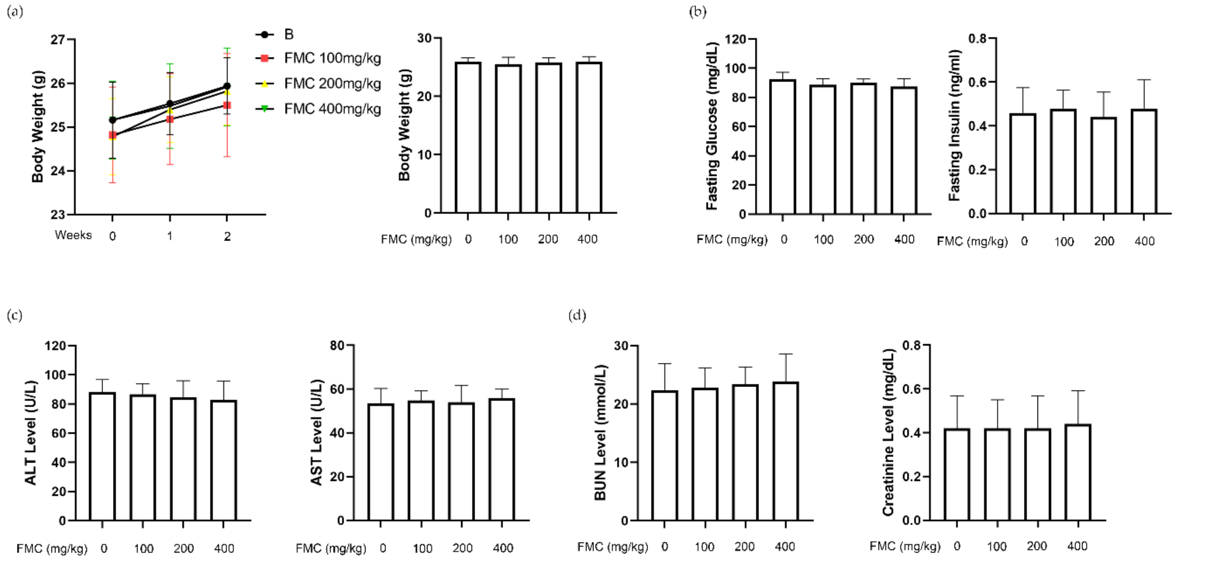

Supplemental Fig 4. Effect of FMC on body weight, fasting blood glucose and insulin and serum parameters in normal mice. FMC was orally administrated for 2 weeks in normal mice. After 2 weeks (a) Body weight of each group, Fasting blood glucose levels and Fasting blood insulin levels (b) BUN, (c) Creatinine, (d) ALT and (e) AST levels in serum in normal mice were measured with or without FMC administration. Results are displayed as mean ± SD of n = 5 per group.

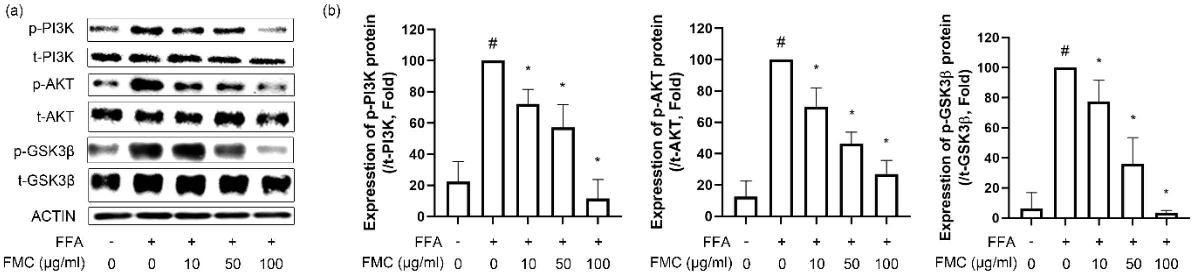

Supplemental Fig 5. Effect of FMC treatment on the IRS-PI3K-AKT pathway in HepG2 cells. Representative pictures show the protein levels of IRS, PI3K, AKT and GSK3β as confirmed by western blot analysis. #$p<0.05$ vs. vehicle-treated cells; *$p<0.05$ vs. FFA-treated cells. Results are displayed as mean ± SD of n = 3 or more.

Supplemental Table 1. Nutritional contents of FMC.

Nutrient compound	Contents	unit
Calories	376.34±0.411	Kcal/100g
Total Carbohydrate	90.57±0.065	g/100g
Total sugars	10.16±0.197	g/100g
Protein	0.95±0.005	g/100g
Total Fat	1.14±0.05	g/100g
Saturated Fat	0.01±0.006	g/100g
Trans Fat	ND	g/100g
Cholesterol	ND	mg/100g
Sodium	1155±4.359	mg/100g
